# Plasminogen Deficiency Significantly Reduces Vascular Wall Disease in a Murine Model of Type IIa Hypercholesterolemia

**DOI:** 10.3390/biomedicines9121832

**Published:** 2021-12-04

**Authors:** Takayuki Iwaki, Tomohiro Arakawa, Mayra J. Sandoval-Cooper, Denise L. Smith, Deborah Donahue, Victoria A. Ploplis, Kazuo Umemura, Francis J. Castellino

**Affiliations:** 1 The W. M. Keck Center for Transgene Research, The Department of Chemistry and Biochemistry, University of Notre Dame, Notre Dame, IN 46556, USA; msandoval@nd.edu (M.J.S.-C.); Denise.L.Smith.804@nd.edu (D.L.S.); Deborah.L.Donahue.16@nd.edu (D.D.); vploplis@nd.edu (V.A.P.); fcastell@nd.edu (F.J.C.); 2 Department of Pharmacology, Hamamatsu University School of Medicine, Hamamatsu 431-3192, Japan; arakawa@hama-med.ac.jp (T.A.); umemura@hama-med.ac.jp (K.U.)

**Keywords:** LDL receptor, apobec-1, plasminogen, atherosclerosis, foam cells

## Abstract

The fibrinolytic system has been implicated in the genesis and progression of atherosclerosis. It has been reported that a plasminogen (Pg) deficiency (*Plg^−/−^*) exacerbates the progression of atherosclerosis in *Apoe^−/−^* mice. However, the manner in which Plg functions in a low-density lipoprotein-cholesterol (LDL-C)-driven model has not been evaluated. To characterize the effect of Pg in an LDL-C-driven model, mice with a triple deficiency of the LDL-receptor (LDLr), along with the active component (apobec1) of the apolipoprotein B editosome complex, and Pg (*L^−/−^/A^−/−^*/*Plg^−/−^*), were generated. Atherosclerotic plaque formation was severely retarded in the absence of Pg. In vitro studies demonstrated that LDL uptake by macrophages was enhanced by plasmin (Pm), whereas circulating levels of LDL were enhanced, relative to *L^−/−^/A^−/−^* mice, and VLDL synthesis was suppressed. These results indicated that clearance of lipoproteins in the absence of LDLr may be regulated by Pg/Pm. Conclusions: The results from this study indicate that Pg exacerbates atherosclerosis in an LDL-C model of atherosclerosis and also plays a role in lipoprotein modification and clearance. Therefore, controlling the Pg system on macrophages to prevent foam cell formation would be a novel therapeutic approach.

## 1. Introduction

Atherosclerosis has been described as a “self-perpetuating” inflammatory disease, which progresses in discrete stages and involves a number of cell types and effector molecules. Upregulation of low-density lipoprotein (LDL) levels, which is the most frequent type IIa hypercholesterolemia, is one of the most important factors causing atherosclerosis in humans. Human liver synthesizes and secretes very low density lipoprotein (VLDL) nascent particles. Each VLDL particle consists of 50–80% triglyceride (TG), 5–15% cholesterol ester (CE), 10–20% phospholipids, and one molecule of apolipoprotein (apo) B100. Nascent VLDL particles in blood acquire apoE and apoCII from high-density lipoprotein (HDL) to form mature VLDL particles. After hydrolysis of TG by lipoprotein lipase and/or hepatic lipase, VLDL is converted to LDL, which in turn delivers CE via LDL receptor (LDLr)-mediated endocytosis [1]. LDLr subsequently binds to the C-terminal region of the single-chain 4536 amino acid residue, apoB100. Amino acid sequences encompassing 3147 to 3157 and 3359 to 3367 of apoB100, which are enriched in basic amino acid residues, have been considered to be major LDLr-binding loci [2]. LDL is extremely susceptible to oxidation (ox) to the more atherogenic ox-LDL, which is not recognized by LDLr, but is taken up by scavenger receptors, such as SR-A1 and CD36 [1].

Spontaneous models of atherosclerosis have emerged through the introduction of genetic mutations in mice. Although the LDLr deficient (*Ldlr^−/−^*) mice and the mice exhibiting deficiency in apoE (*Apoe^−/−^*) are heavily used for these studies, *Apoe^−/−^* and *Ldlr^−/−^* mice that are on a normal chow diet do not mimic type IIa hypercholesteroremia [3,4,5]. Murine hepatocytes express Apolipoprotein B Editing Enzyme 1 (*Apobec1*) that alters cytosine in the first base of codon 2153 of *Apob* mRNA to uracil. Therefore, codon 2153 is changed to “UAA” from “CAA”, and murine liver secretes truncated apoB48 (2152 amino acids) containing VLDL. Its expression level increases after birth. The livers of mature 2 month-old mice synthesize VLDL, containing 70% apoB48 and 30% apoB100 [6]. Therefore, LDL in *Ldlr^−/−^* mice does not increase significantly, unlike in humans. LDLr is not heavily involved in the clearance of LDL in this mode. It has been shown that a mouse model of elevated LDL is one in which both the *Ldlr* and *Apobec1* genes are deleted (*L^−/−^/A^−/−^*). This is due to the fact that these mice lack the ability to convert apoB100 to apoB48 in the liver and are defective in LDL clearance [7]. These doubly deficient mice slowly and progressively present with severe spontaneous atherosclerosis on a normal chow diet. They also exhibit high levels of apoB100-LDL-containing hypercholesterolemia, and more closely mirror the plasma lipid profiles in human atherosclerosis [8].

Plasminogen (Pg), which is encoded by the *PLG* gene in humans, is the zymogen of the serine protease, plasmin (hPm). Human Pg is a 92 kDa single chain glycoprotein that is activated by Pg activators (PA), e.g., tissue-type plasminogen activator (tPA) and urokinase-type plasminogen activator (uPA) [9], via the cleavage of a single peptide bond at Arg^561^-Val^562^, leading to the disulfide-linked, two-chain hPm. The 561 amino acid N-terminal heavy chain contains five kringle domains, four of which bind to Lys-residues in fibrin [10], and other proteins, e.g., Pg receptors, in prokaryotes [11] and eukaryotes [12,13]. The potential importance of the fibrinolytic system in regulating atherosclerosis is highlighted by the observations of fibrin and fibrin degradation products in atherosclerotic plaques [14]. Mice with alteration in the gene-encoding components of the fibrinolytic system are powerful tools to evaluate the relationships between atherosclerosis and fibrin clearance. One study employing such mice indicated that Pg deficiency greatly accelerated the development of atherosclerotic lesions in *Apoe^−/−^* mice without altering total cholesterol (TC) levels. The authors showed that Pg deficiency did not block the progression of lesions to any specific stage because mice of both genotypes carried intimal lesions. These had the qualitative appearance of early, intermediate, and advanced disease. The lesions included fibrous plaques containing necrotic cores, cholesterol clefts, macrophage foam cells, smooth muscle cells, and deposits of elastic fibers and collagen [15]. On the other hand, proteolytic cleavage of apoB100 by trypsin or plasmin (Pm), in conjunction with hydrolysis of cholesteryl esters, generated lipoprotein particles that were similar to lesions derived from human LDL in structure, biological properties, and composition [16,17]. Further, Pm modified human LDLs were efficiently taken up by human macrophages [18,19]. Based on these results, we hypothesized that Pg binds to the Lys-rich C-terminus of apoB100, and Pm-mediated apoB100-modified LDL particles are efficiently taken up by macrophages. This reaction is significantly reduced by the presence of *Apobec1* in *Apoe^−/−^* mice.We have generated *L^−/−^/A^−/−^*/*Plg^−/−^* mice and characterized the progression of plaque formation in these mice in order to test this hypothesis.

## 2. Materials and Methods

### 2.1. Mice

Plasminogen deficient mice (*Plg^−/−^*) and *L^−/−^*/*A^−/−^* mice were back-crossed into the C57Bl/6J strain (Jackson Laboratory, Bar Harbor, ME, USA) for at least seven generations before cross-breeding [7,8,20,21,22]. Triple heterozygous (*L^+/−^/A^+/−^/Plg^+/−^*) mice were crossed to *L^−/−^/A^−/−^* to produce the *L^−/−^/A^−/−^/Plg^+/−^* as breeders. The primers and probes used for genotyping are listed in Supplementary Table S1. Male mice were used in in vivo experiments and both genders were used in in vitro experiments. Mice were maintained on a standard chow diet for 12–24 wk. Mice were fasted for at least 6 h and sacrificed at each time point using Isoflurane (Abbvie, North Chicago, IL, USA). The blood obtained was anticoagulated with sodium citrate, EDTA, or heparin. The hearts and whole aortic trees were removed for morphometrical analyses after perfusion with isotonic saline.

### 2.2. Complete Blood Count (CBC) and Plasma Lipid Analysis

Total white blood cell, platelet, monocyte, lymphocyte, and neutrophil counts were performed on EDTA-treated blood from all genotypes at 12 wk, using a Hemavet 950F5 (Drew Scientific Inc., Waterbury, CT, USA). Plasma was separated from whole citrated blood and utilized to measure TC using Cholesterol CII (Wako Chemicals, Richmond, VA, USA), and TG using GPO-TRINDER (MilliporeSigma, St. Louis, MO, USA) [8,23].

### 2.3. FPLC Analysis for Cholesterol Fractions

Cholesterol profiles were examined using 100 µL of plasma with fast performance liquid chromatography (FPLC) on a Superose 6 HR (Amersham Pharmacia biotech, Piscataway, NJ, USA), as previously reported [8,23].

### 2.4. Heart Sections

Hearts were cut at the level of the lower edge of the atrium. The upper part, containing the aortic valve, was fixed with periodate-lysine-paraformaldehyde (PLP) for 16 h at 4 °C. After fixation, some samples were directly embedded in Tissue-Tec OCT (Sakura Fine Tec Co., Torrance, CA, USA). Other samples were processed and embedded in paraffin, cut side down. Thirty serial sections (from #1 to #30) were obtained at 4 µm thickness from the aortic valve toward the ascending aorta, as previously described [8,23].

### 2.5. Histochemistry and Immunohistochemistry

Serial sections were used for hematoxylin II and eosin Y (H&E) (Richard Allen Scientific, Kalamazoo, MI, USA). The sections were also stained in oil red O (ORO; Sigma-Aldrich, St. Louis, MO, USA) and Masson’s trichrome in order to detect lipid accumulation in the plaque for the determination of the size of the plaque in the aortic bulb, as previously described [8,23]. Immunohistochemical staining of macrophages was performed with rat-anti-mouse MAC3 and F4/80 cocktail (BD Pharmingen and AbD Serotec MorphoSys US, Raleigh, NC, USA, respectively). The stained slides were digitally scanned at 20× magnification with an Aperio CS digital slide scanner (Aperio Technologies, Vista, CA, USA).

### 2.6. Assessment of Hepatic VLDL-TG Production

After a 6 h fasting period, mice were anesthetized by i.p. administration of 0.075 mg/g ketamine/0.015 mg/g xylazine/0.0025 mg/g aceprozamine. In order to block TG hydrolysis and hepatic lipoprotein uptake, 500 µg/g of Tyloxapol (Triton WR-1339; Fisher Scientific, Hampton, NH, USA) was injected via the tail vein [24]. Blood samples (30 µL) were withdrawn at 0, 30, 60, 90, and 120 min after injection, and plasma TG levels were measured, as described above. The VLDL-TG production rates were then calculated.

### 2.7. Culture of Bone Marrow-Derived Macrophages

Bone marrow was collected from the femurs of *L^−/−^/A^−/−^* and *L^−/−^/A^−/−^/Plg^−/−^* mice at 8–12 wk. Bone marrow-derived macrophages were generated. This was performed by culturing non-adherent bone marrow cells with RPMI 1640 medium (MilliporeSigma), supplemented with 20% FCS/30% L-cell conditioned medium (as the source of M-CSF)/antibiotic/antimycotic solution (100 U/mL of penicillin G, 100 µg/mL of streptomycin, and 250 ng/mL of amphotericin B; Sigma-Aldrich) for 7 days. Most cells were adherent to the dishes, and floating cells were removed by washing twice with warm HBSS (Millipore Sigma). The adherent bone marrow-derived macrophages were incubated with ice cold sterile HBSS for 15 min at 4 °C to detach, and the cells were then collected.

### 2.8. DiI-Oxldl Uptake by Bone Marrow-Derived Macrophages

The collected bone marrow-derived macrophages as above were resuspended in the conditioned media at 5.0 × 10^6^ cells/mL. The 100 μL cell suspension (5.0 × 10^5^ cells) was placed into each well of a 96-well plate, and the cells were incubated at 37 °C in a 5% CO_2_ incubator for 16 h to settle. The media were removed, and the cells were washed twice with warm HBSS. The cells were incubated with 100 μL RPMI1640 containing DiI-oxLDL (20 µg/mL; Biomedical Technologies) at 37 °C in a 5% CO_2_ incubator for 5 h. The DiI was subsequently extracted from the cells using 100 μL of 2-propanol. The fluorescent intensity was measured using an ABI prism 7700 (Applied Biosystems, Foster City, CA, USA) using the Joe channel (absorption at 520 nm and emission at 548 nm). Various concentrations of murine wild-type (wt) Pg and an active site mutant [S^743^A]-Pg recombinantly synthesized by S2 cells, which are derived from *Drosophila melanogaster*, without bovine serum [25], along with aprotinin (USB Corporation, Cleveland, OH, USA), were added to the culture to evaluate the functions of Pg and Pm. We have chosen Drosophila S2 cells without fetal bovine serum, as opposed to mammalian cell lines, to express recombinant Pg because of the ubiquitous presence of hPg activators in mammalian cells.

### 2.9. RNA Sequencing

Total RNA was extracted from the livers of the *L^−/−^/A^−/−^* and *L^−/−^/A^−/−^/Plg^−/−^* mice using the RNeasy Mini Kit (Qiagen, Valencia, CA, USA). The total RNA quality was evaluated using a NanoDrop 1000 (Thermo Fisher Scientific, Waltham, MA, USA), and the RNA integrity of the samples was assessed. All samples passed the quality checks. Libraries were constructed using a TruSeq Stranded mRNA LT Sample Prep Kit (Illumina, San Diego, CA, USA) and sequenced on the Illumina platform. Raw data was trimmed using Trimmomatic 0.38 (www.usadellab.org/cms/?page=trimmomatic (accessed on 19 March 2020)). Trimmed data was mapped to a reference genome (mm10) using HISAT2 v. 2.1.0 and Bowtie2 v. 2.3.4.1 (ccb.jhu.edu/software/hisat2/index.shtml (accessed on 19 March 2020)). Differentially expressed genes (DEGs) were identified using StringTie v. 2.1.3b (ccb.jhu.edu/software/stringtie/ (accessed on 19 March 2020)). The significance threshold was *p* < 0.05. The fold change was >2 or <0.5 and this was used to identify DEGs. Gene Ontology (GO) enrichment analysis and Kyoto Encyclopedia of Genes and Genomes (KEGG) pathway enrichment analysis of the DEGs were conducted. These processes were supported by Macrogen Japan Corp (Tokyo, Japan).

### 2.10. Fluorescence-Assisted Cell Sorting (FACS) of Macrophages in Lever

FACS was performed as described in [26]. The mice were briefly perfused with EGTA (ethylene glycol tetra-acetic acid)/HBSS (Hank’s balanced salt solution)/collagenase (Sigma-Aldrich). After filtration through a cell strainer, the cells were collected using percoll. The resulting samples were stained with antibodies against CD45, CD11b, and F4/80 and sorted using a MoFlo Astrios (Beckman Coulter, Brea, CA, USA) for mRNA expression analysis.

### 2.11. Quantitative Real-Time PCR

Total RNA was extracted as above from livers and hepatocytes, as well as the sorted cells. Reverse transcription was performed using a ReverTra Ace qPCR RT Master Mix with gDNA Remover (TOYOBO, Tokyo, Japan). Quantitative, real-time PCR was performed using the THUNDERBIRD SYBR qPCR Mix (TOYOBO) and QuantStudio 3 (Thermo Fisher Scientific). The CD36 primer sequences were 5′-CAAAACGACTGCAGGTCAAC-3′ (forward) and 5′-CCAATGGTCCCAGTCTCATT-3′ (reverse). The 18S rRNA primer sequences were 5′-GGTAACCCGTTGAACCCCAT-3′ (forward) and 5′-CAACGCAAGCTTATGACCCG-3′ (reverse). The results were expressed as the mRNA level relative to 18S rRNA mRNA as an internal control.

### 2.12. Statistical Analyses

The data are presented as the mean ± SD. The size of plaques and plasma levels of Total-C, VLDL-C, LDL-C, and HDL-C, triglyceride, de novo hepatic synthesis of VLDL, sP-Sel and sE-sel, and mRNA levels were analyzed using Student’s t-test for each time point. The DiI-oxLDL uptakes were analyzed using one-way factorial ANOVA, followed by Scheffe. All analyses were performed using the R Statistical Computing Package (R Developmental Core Team, http://www.R-project.org (accessed on 14 May 2020)), and null hypothesis was declined at *p* < 0.05.

## 3. Results

### 3.1. Plaque Progression in Whole Aorta and Aortic Sinus

**Figure 1:**
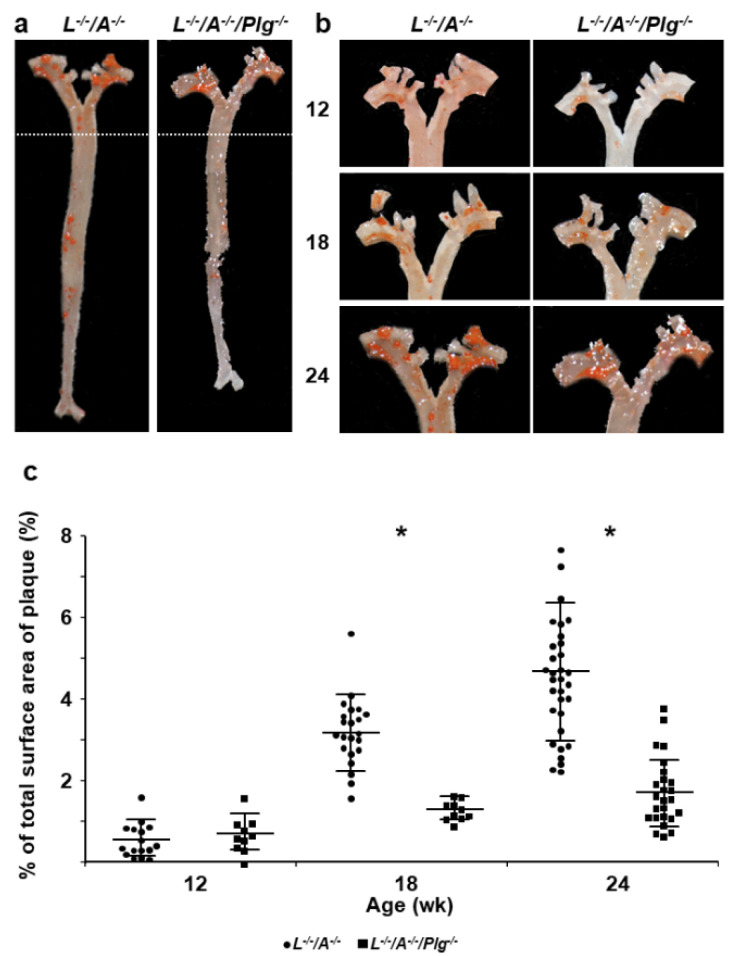
Plaque formation in entire aortic trees. (**a**) Sudan IV staining of whole aortic trees in *L^−/−^/A^−/−^* and *L^−/−^/A^−/−^/Plg^−/−^* mice at 24 wk of age. (**b**) Sudan IV staining of whole aortic arches in *L^−/−^/A^−/−^* and *L^−/−^/A^−/−^/Plg^−/−^* mice at 12, 18, and 24 wk of age. (**c**) The percentage of total surface occupied by plaque in the aorta of *L^−/−^/A^−/−^* mice was 0.56 ± 0.41, 3.1 ± 0.91, and 4.6 ± 1.7 at 12, 18, and 24 wk of age, respectively (circles). That in *L^−/−^/A^−/−^/Plg^−/−^* mice was 0.69 ± 0.44, 1.3 ± 0.24, and 1.7 ± 0.84 at 12-, 18-, and 24 wk of age, respectively (squares), (*n* = 10–58). * *p* < 0.05.

**Figure 2:**
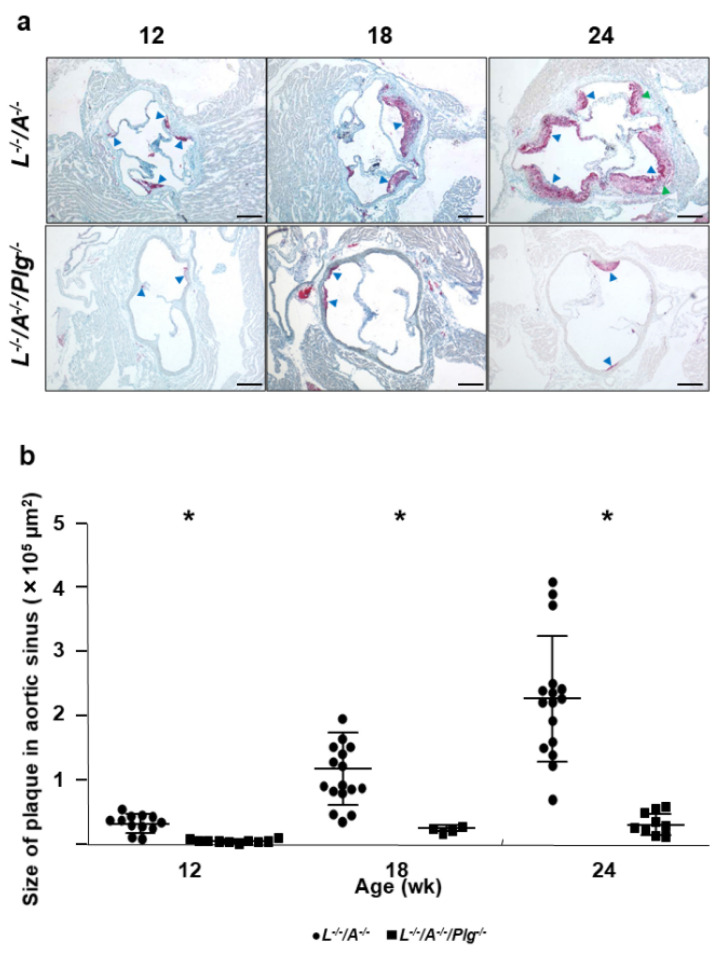
(**a**) Oil red O (ORO) stains for lipids associated with the aortic sinuses from *L^−/−^/A^−/−^* and *L^−/−^/A^−/−^/Plg^−/−^* male mice at various ages. In 12 wk *L^−/−^/A^−/−^* mice, there were dense droplets of lipids associated with the base of aortic valves (blue arrowheads). In 12 wk *L^−/−^/A^−/−^/Plg^−/−^* mice, the area of fat accumulation was smaller than in *L^−/−^/A^−/−^* (blue arrowheads). In 18 wk *L^−/−^/A^−/−^* mice, the neointima was clearly observed with dense fat accumulation (blue arrowheads). In 18 wk *L^−/−^/A^−/−^/Plg^−/−^* mice, the area of fat accumulation was enhanced relative to 12 wk mice although neointima formation was significantly restricted compared with *L^−/−^/A^−/−^* at the same age (blue arrowheads). In 24 wk *L^−/−^/A^−/−^* mice, the neointima was enhanced (blue arrowheads). The lipid stains in the neointima became less dense particularly in the core due to the formation of complex plaque (green arrowheads). In 24 wk *L^−/−^/A^−/−^/Plg^−/−^* mice, small and restricted neointima formation was observed (blue arrowheads). Original magnification, 40×. Bar = 250 µm. (**b**) Plaque sizes in aortic sinuses of *L^−/−^/A^−/−^* were 0.33 ± 0.15, 1.2 ± 0.57, and 2.3 ± 0.93 at 12, 18, and 24 wk of age, respectively (circles). Those in *L^−/−^/A^−/−^/Plg^−/−^* mice was 0.046 ± 0.026, 0.23 ± 0.045, and 0.31 ± 0.17 at 12, 18, and 24 wk of age, respectively. Those in *L^−/−^/A^−/−^/Plg^−/−^* (squares) mice, as revealed by morphometric analysis of ORO-stained slides (*n* = 4–16). * *p* < 0.05.

The plaque covered area in whole aorta (Figure 1) and the plaque size in aortic sinuses (Figure 2) were analyzed. The area and the size were increased from 12, 18, and 24 wk of age in *L^−/−^/A^−/−^* mice (Figure 1 and Figure 2). The plaques were only visible in the aortic arches of mice at 12 wk of age, and were conspicuous around the intercostal and celiac arteries at 24 wk of age (Figure 1a). This increase continued through 72 wk of age in *L^−/−^/A^−/−^* mice [8]. On the other hand, those in *L^−/−^/A^−/−^/Plg^−/−^* mice were more significantly reduced at 12, 18, and 24 wk of age than the plaques in *L^−/−^/A^−/−^* mice (Figure 1 and Figure 2). These results indicated that the rate of progression of plaques was restricted, and the initiation of plaque formation was delayed.

### 3.2. Histological and Immunohistological Characterization of Plaques

**Figure 3:**
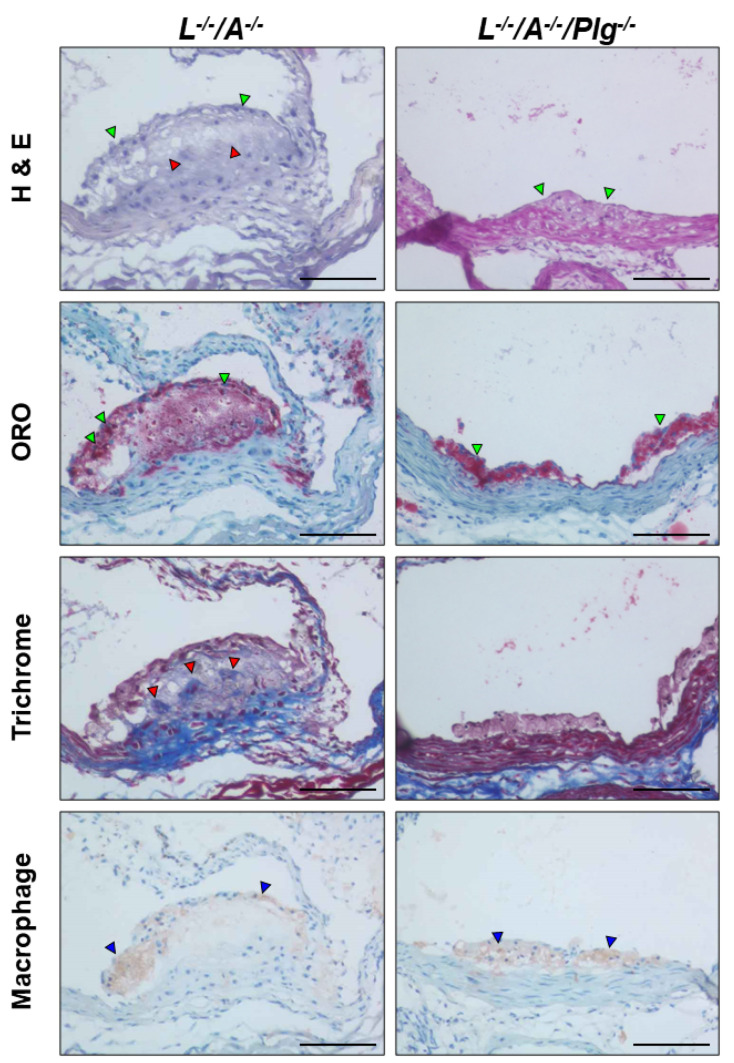
Stained plaque from *L^−/−^/A^−/−^* and *L^−/−^/A^−/−^/Plg^−/−^* male mice at 18 wk of age. H&E stains in *L^−/−^/A^−/−^* demonstrated cellular cap formation on the surface of the plaque (green arrowheads) with acellular areas (red arrowheads). H&E stains in *L^−/−^/A^−/−^/Plg^−/−^* mice indicated that the size of plaque was very small and only a few cell layers were observed (green arrowheads). ORO stains in *L^−/−^/A^−/−^* mice demonstrated that the dense fat accumulation was mainly localized on the surface of the plaque (green arrowheads). Fat accumulation was diffuse in the core. ORO stains in *L^−/−^/A^−/−^/Plg^−/−^* mice demonstrated small but very dense fat accumulation in the plaque (green arrowheads), which was found earlier in *L^−/−^/A^−/−^* mice. Masson’s trichrome stains in *L^−/−^/A^−/−^* mice showed diffuse and patchy collagen deposition (red arrowheads). Masson’s trichrome stains in *L^−/−^/A^−/−^/Plg^−/−^* mice indicated an absence of collagen deposition in the plaque. Macrophage immunostains in *L^−/−^/A^−/−^* mice showed positive stains mainly localized in the cap area (blue arrowheads). Macrophage immunostains in *L^−/−^/A^−/−^/Plg^−/−^* mice indicated that almost all of the cells were positive in the small cap (blue arrowheads). Original magnification, 200×. Bar = 100 µm.

H&E stains from 18 wk old mice demonstrated that *L^−/−^/A^−/−^* mice partially developed a fibrous cap on the surface of plaque. Notwithstanding, *L^−/−^/A^−/−^/Plg^−/−^* mice contained very small plaques at this stage. ORO stains demonstrated that both sets of mice contained lipid-accumulated plaques; although, the sizes of the plaques in *L^−/−^/A^−/−^/Plg^−/−^* mice were much smaller than those in *L^−/−^/A^−/−^/Plg^−/−^* mice. Trichrome stains revealed a collagen core associated with SMCs in *L^−/−^/A^−/−^* mice. In contrast, neither collagen deposits nor SMCs were found in plaques of *L^−/−^/A^−/−^/Plg^−/−^* mice. Macrophage immunostains were positive in plaques in both genotypes. However, the positive stains in plaques of *L^−/−^/A^−/−^* mice were rarely associated with nuclei, in contrast to the positive stains associated with nuclei in plaques in *L^−/−^/A^−/−^/Plg^−/−^* mice (Figure 3). These results indicated that the reduced progression of plaques in *L^−/−^/A^−/−^/Plg^−/−^* mice was correlated to the foam cell formation of macrophages.

### 3.3. Lipid Analysis and De Novo Hepatic Synthesis of VLDL

**Figure 4:**
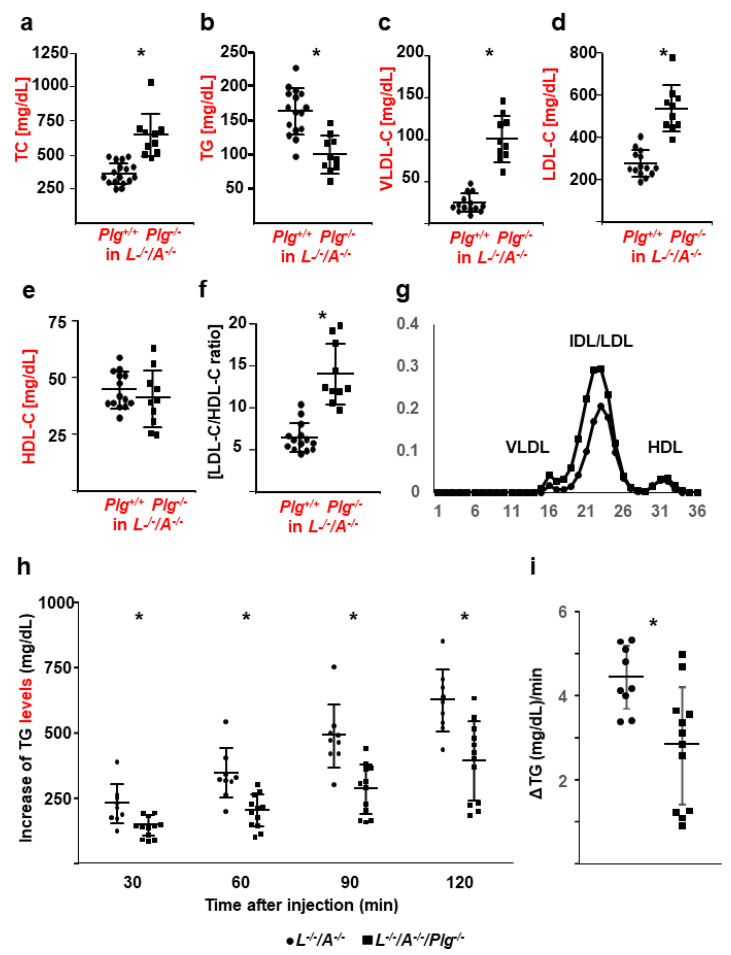
Lipid profiles, CBC, and De novo hepatic synthesis of VLDL. (**a**) TC, (**b**) TG, (**c**) VLDL-C, (**d**) LDL-C, (**e**) HDL-C, and (**f**) LDL-C/HDL-C in *L^−/−^/A^−/−^* (circles) and *L^−/−^/A^−/−^/Plg^−/−^* (squares) mice (*n* = 8–19). (**g**) Typical FPLC profile. (**h**) Increase in plasma TG as de novo VLDL synthesis in *L^−/−^/A^−/−^* (circles) and *L^−/−^/A^−/−^/Plg^−/−^* (squares) mice (*n* = 9–12). (**i**) The VLDL production rate was calculated. * *p* < 0.05.

The values of TC, TG, VLDL-C, LDL-C, HDL-C, and LDL-C/HDL-C are summarized in Figure 4. The plasma levels of TC, VLDL-C, LDL-C, and LDL-C/HDL-C in *L^−/−^/A^−/−^/Plg^−/−^* mice were significantly higher than those in *L^−/−^/A^−/−^* mice (Figure 4a–d,f, respectively). The plasma levels of TG in *L^−/−^/A^−/−^/Plg^−/−^* mice were significantly lower than those in *L^−/−^/A^−/−^* mice (Figure 4b). However, there were no significant differences in HDL-C levels (Figure 4e). In contrast, *Apoe^−/−^/Plg^−/−^* mice showed down-regulated HDL-C without changing TC [15]. TG levels in both *L^−/−^/A^−/−^/Plg^−/−^* and *Apoe^−/−^/Plg^−/−^* mice were diminished. Since *Plg^−/−^* mice showed recto-prolapse and the body weight was decreased after developing it, we have speculated that the TG levels reflected to the food intake. De novo hepatic synthesis of VLDL-TG was more significantly reduced in *L^−/−^/A^−/−^/Plg^−/−^* mice than in *L^−/−^/A^−/−^* mice (Figure 4g,h); thus, clearly indicating that up-regulation of LDL-C was not initiated by over synthesis of VLDL.

### 3.4. OxLDL Uptake by Macrophages Is Diminished with a Pg Deficiency

**Figure 5:**
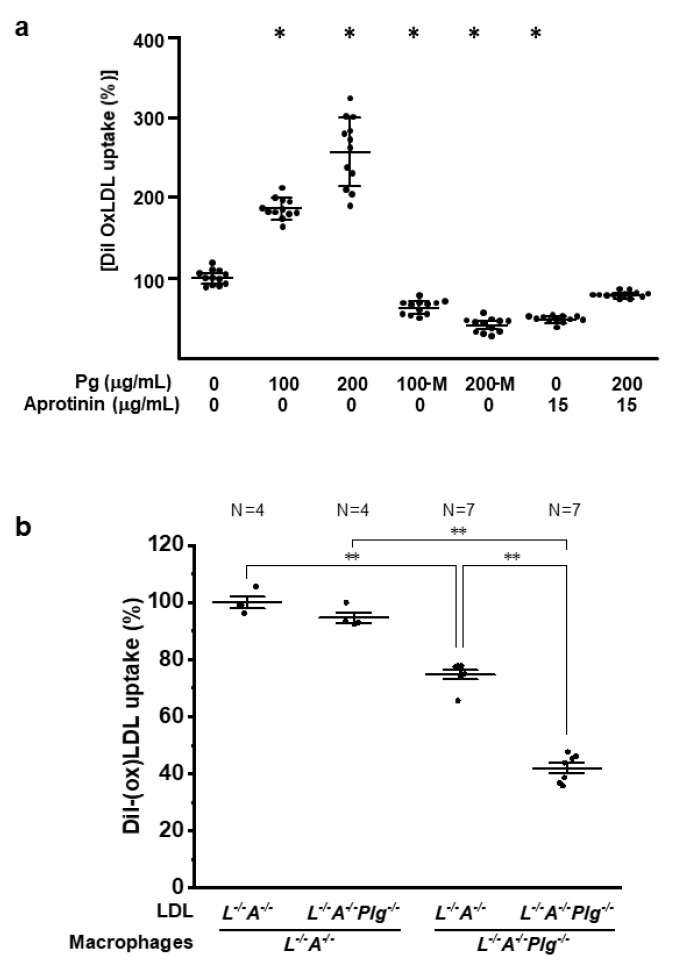
LDL/oxLDL uptake. (**a**) Purified and Dil-labeled oxLDL was incubated with bone marrow-derived macrophages from 12 wk *L^−/−^/A^−/−^* mice (*n* = 12), in the absence or presence of increasing concentrations of Pg and in the absence or presence of the plasmin inhibitor, aprotinin. M—active site mutant Pg protein (PgS743A). * *p* < 0.05 versus (-) mPg and (-) aprotinin. (**b**) LDL was isolated from *L^−/−^/A^−/−^* and *L^−/−^/A^−/−^/Plg^−/−^* mice, Dil-labeled, and then incubated with bone marrow-derived macrophages from *L^−/−^/A^−/−^* (circles) or *L^−/−^/A^−/−^/Plg^−/−^* (squares) mice at 12 wk of age. Macrophages isolated from 12 wk mice and LDL obtained from 12 wk mice (*n* = 4 for circles and *n* = 7 for squares) * *p* < 0.05. “*” is “**” between the pairs of comparisons.

After it was determined that there were differences in the oxidation status of plasma between the two mouse genotypes, an analysis of the efficiency of uptake of oxLDL by macrophages was carried out. Bone marrow-derived macrophages from *L^−/−^/A^−/−^* mice were incubated with increasing concentrations of Pg in the presence and absence of the hPm inhibitor, aprotinin. Dil-labeled human oxLDL uptake increased with the addition of Pg in a dose-dependent manner and uptake was virtually abolished with the addition of aprotinin, or through the use of a recombinant Pg-active site mutant (PgS743A) (Figure 5a). The in vitro uptake of LDL by macrophages from *L^−/−^/A^−/−^* and *L^−/−^/A^−/−^/Plg^−/−^* was assessed by FPLC of LDL, isolated from *L^−/−^/A^−/−^* and *L^−/−^/A^−/−^/Plg^−/−^* mice. Uptake of LDL was the least efficient in bone marrow-derived macrophages and LDL from *L^−/−^/A^−/−^/Plg^−/−^* mice (Figure 5b). These results indicated that Pm-mediated LDL was more atherogenic and the uptake of oxLDL by macrophages was dependent Pm.

### 3.5. RNA Sequencing and the Expression of Cd36

**Figure 6:**
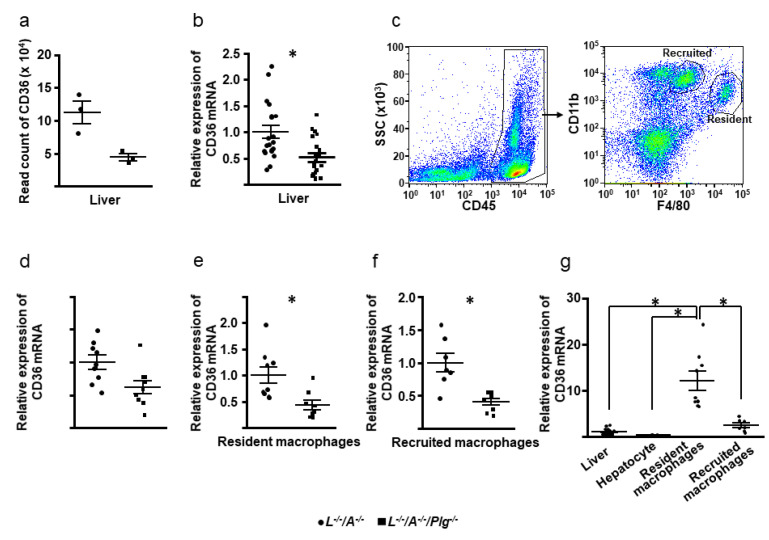
(**a**) Read count of *Cd36* by RNA sequencing (*n* = 3). (**b**) The mRNA expression levels of *Cd36* in liver (*n* = 20). (**c**) CD45+ cells were analyzed with F4/80 and CD11b, and resident (F4/80hi) and recruited (CD11bhi) macrophages were sorted. (**d**–**f**) The mRNA expression levels of *Cd36* in hepatocytes and recruited and resident macrophages (*n* = 7–9). (**g**) Comparison of the mRNA expression levels of *Cd36* in liver, hepatocytes, and recruited and resident macrophages in *L^−/−^/A^−/−^* mice (*n* = 7–20). * *p* < 0.05 between *L^−/−^/A^−/−^* and *L^−/−^/A^−/−^/Plg^−/−^* in (**b**,**e**,**f**). In (**g**), between the pairs of comparisons.

We conducted sequencing of RNA from the liver, since the liver plays a key role in lipid metabolism. In total, 753 differentially expressed genes (DEGs) were identified using the specified selection criteria (*p* < 0.05; fold change >2 or <0.5). Of these, expression levels of 337 were upregulated while those of 416 were downregulated in *L^−/−^/A^−/−^/Plg^−/−^* mice compared with *L^−/−^/A^−/−^* mice. After checking the profiles by comparing expression of scavenger receptors and genes related to lipid metabolisms, it was clearly confirmed that *Cd36* expression in liver was lower in *L^−/−^/A^−/−^/Plg^−/−^* mice than in *L^−/−^/A^−/−^* mice (Figure 6a). To validate these RNA sequencing results, we performed q-RTPCR on 40 liver samples, including the RNA sequencing on 6 samples. These PCR results revealed lower *Cd36* expression in *L^−/−^/A^−/−^/Plg^−/−^* mice than in *L^−/−^/A^−/−^* (Figure 6b). Then, macrophages were sorted from livers in order to analyze the expression of *Cd36* in macrophages (Figure 6c). Macrophages were subsequently sorted from livers in order to analyze the expression of *Cd36* in macrophages (Figure 6c). A recently developed FACS protocol enabled distinction between resident and recruited macrophages [26]. As shown in Figure 6d–f, the mRNA expression levels of *Cd36* in hepatocytes showed that recruited and resident macrophages were all reduced in *L^−/−^/A^−/−^/Plg^−/−^* compared with *L^−/−^/A^−/−^*. Interestingly, the mRNA expression level of *Cd36* in resident macrophages was significantly higher than that of liver, hepatocytes, and recruited macrophages (Figure 6g).

## 4. Discussion

It is a known medical fact that *Apoe^−/−^* mice are characterized as a VLDL-C dominant spontaneous atherosclerosis model. On the other hand, *L^−/−^/A^−/−^* mice are characterized as an LDL-C dominant model [7,8]. Even though high levels of both VLDL-C and LDL-C could initiate and propagate atherosclerotic plaques, the processes involved in plaque formation by different types of hypercholesterolemia differ. Indeed, VLDL remnants could trigger foam cell formation from monocytes/macrophages via VLDLr-mediated uptake, without oxidization [27]. However, such oxidization is critical for LDL to initiate foam cell formation [28,29,30]. Furthermore, VLDL can bind to coagulation factors (F)II, FVII, FIX, and FX, which are Vitamin K-dependent proteins containing g-carboxyglutamate (Gla) domains that facilitate binding to phospholipids. On the other hand, binding of these proteins to LDL has not been documented [31]. Only one molecule of apoB100 is present in a particle of VLDL/IDL/LDL, but the surface of human LDL is almost completely covered by apoB100 [2]. Thus, there are few phospholipids on LDL available to bind these proteins. Additionally, the Lys-rich C-terminus of apoB100 in LDL is a potential target of Pg binding, followed by proteolytic degradation, and this reaction is reduced in mice due to the presence of *Apobec1* in their livers. These considerations indicate that alterations in the coagulation and fibrinolytic systems, induced by hypercholesterolemia, may be dependent on the types and/or components of lipoproteins. Therefore, we developed the lipid profiles of *L^−/−^/A^−/−^/Plg^−/−^* mice and analyzed them in the current study.

In this study, an additional Pg deficiency in *L^−/−^/A^−/−^* mice upregulated plasma levels of TC, VLDL-C, and LDL-C, but did not affect HDL-C levels, and de novo VLDL synthesis was impaired in *L^−/−^/A^−/−^/Plg^−/−^* mice. Thus, upregulation of LDL-C was the result of reduced clearance of this lipid particle. Although hypercholesterolemia, especially in the LDL-C class, was observed in *L^−/−^/A^−/−^/Plg^−/−^* mice, the sizes of the plaques were dramatically reduced, and the progression of plaque development was retarded. These results were in contrast to those obtained with *Apoe^−/−^/Plg^−/−^* mice [15].

Monocyte infiltration to the plaques was enhanced in *Apoe^−/−^/Plg^−/−^* mice [15] and this phenomenon was also detected in *L^−/−^/A^−/−^/Plg^−/−^* mice. Moreover, monocyte recruitment into the liver of both *L^−/−^/A^−/−^* and *L^−/−^/A^−/−^/Plg^−/−^* mice was not different. It is well known that the vascular endothelial structure in BM and the liver is a perforated structure. The numbers of WBCs in the blood were highly elevated in *Plg^−/−^* mice [20] as well as in *L^−/−^/A^−/−^/Plg^−/−^* mice (data not shown). Taken together, the transfer of WBCs through a specific endothelium was demonstrated to be unaffected by Pg deficiency. This was probably because this pathway did not require the use of extracellular matrix proteolysis. Therefore, macrophage infiltration from damaged vascular endothelial cells of the aorta into the subendothelial cell layer may be unaffected by Pg deficiency.

In contrast, oxLDL uptake by macrophages from *L^−/−^/A^−/−^* mice was Pg dose-dependent and decreased with the addition of active site-inactivated Pg or Pm inhibitors. Additionally, uptake of (ox)LDL from *L^−/−^/A^−/−^/Plg^−/−^* mice by macrophages from *L^−/−^/A^−/−^/Plg^−/−^* mice was significantly reduced compared with *L^−/−^/A^−/−^* mice. These results indicated that Pm activity played an important role in the modification of LDL and the uptake of (ox)LDL by macrophages, although there was a possibility that the results might be due to oxidized phospholipids because Pg in the circulation in vivo was a sink for oxidized phospholipids. Furthermore, higher plasma levels of TC, VLDL-C, and LDL-C were not triggered by hyper-hepatic synthesis of VLDL, which strongly implies that LDL metabolism itself was also downregulated by a Pg deficiency.

The major apolipoprotein in LDL is apoB100 in humans. Trypsin and Pm were able to cleave apoB100 in LDL. Consequently, small peptides were released from the LDL particle [32,33,34]. These enzymatically modified LDLs (ELDL) induced foam cell formation in macrophages [17], stimulated MCP-1 production [35], and promoted the adhesion of monocytes to the endothelial cell layer [36]. Moreover, ELDL formed by Pm was taken up more readily by human macrophages than murine macrophages [18].

There are four SRs that bind to modified LDL identified in macrophages [37]. Among them, SR-AI/II and CD36 are thought to be important for uptake of modified LDL [38]. In this study, RNA sequencing revealed that *Cd36* expression by liver in *L^−/−^/A^−/−^/Plg^−/−^* mice was diminished. It is thought that most of the signal was derived from hepatocytes. In the liver, there were two types of macrophages, namely, recruited and resident. *Cd36* expression in both cell types was also diminished in *L^−/−^/A^−/−^/Plg^−/−^* mice. Interestingly, resident macrophages expressed more *Cd36* than recruited macrophages, so macrophage might respond differently to the uptake of modified LDL, depending on the stage of differentiation. The downregulation of *Cd36* in *Apoe^−/−^/Plg^−/−^* mice has also been previously reported in the literature [39]. The same authors also reported that foam cell formation of the macrophages prepared from *Apoe^−/−^/Plg^−/−^* mice by human oxLDL was diminished [39]. However, this could be contradictory to the initial characterization of the mice [15]. Moreover, it was reported that loss of receptor-mediated lipid uptake via SR-AI/II or CD36 pathways did not ameliorate atherosclerosis. It was also reported that it did not ameliorate loss of SR-AI/II, and CD36 did not abrogate foam cell formation in *Apoe^−/−^* mice [40,41]. Preparation of murine LDL is difficult and human LDL/oxLDL is used in in vitro experiments with murine macrophages in many cases. We have speculated that the interaction between the C-terminus of apoB100 and Pg is key to explain some discrepancies in *Apoe^−/−^* mice experiments. Consequently, we have developed *L^−/−^/A^−/−^/Cd36^−/−^* and *L^−/−^/A^−/−^/Msr1^−/−^* mouse lines and are now evaluating them. Moreover, it was reported that the gene expression levels of *Cd68* (related to lipid uptake), *Abca1* (related to phagocytosis), and *Pparg* (related to nuclear receptors) in *Apoe^−/−^/Plg^−/−^* mice were significantly more reduced than those in *Apoe^−/−^* mice [39]. However, those in macrophages from *L^−/−^/A^−/−^* and *L^−/−^/A^−/−^/Plg^−/−^* mice were not significantly different.

In conclusion, we showed that Pg/Pm accelerated foam cell formation in an apoB100-positive, LDL-C-dominant murine model by enhancing uptake of oxLDL, likely modifying apoB100. The most common hypercholesterolemia in humans is type IIa, in which LDL containing apoB100 is dominant. The methods of preventing foam cell formation are clinically important, but this idea has not been put to practical use to treat atherosclerosis in humans. More recently, it was reported that there is a novel Pg receptor—Plg-R_KT_—which is an integral membrane protein that exposes a C-terminal Lys residue on this receptor in macrophages [42,43], and that Pg on macrophages accelerated wound healing [44]. Furthermore, overexpression of uPA in macrophages of *Apoe^−/−^* mice resulted in acceleration of atherosclerosis [45], and cholesterol efflux from macrophages was upregulated by Pg [46]. Therefore, not only scavenger receptors, but also their ligands and other receptors related to Pg, as well as Pg activation system on macrophages, need to be investigated in *L^−/−^/A^−/−^* mice.

## Data Availability

The data presented in this study are available on request from the corresponding author.

## Supplementary Materials

The following are available online at https://www.mdpi.com/article/10.3390/biomedicines9121832/s1, Table S1: Primers and probes for genotyping of interbred mice with combined deficiencies of *Ldlr*, *Apobec1*, and *Plg.*
