# Supplementary material for: Plasminogen Deficiency Significantly Reduces Vascular Wall Disease in a Murine Model of Type IIa Hypercholesterolemia
Source: Biomedicines. 2021 Dec 4;9(12):1832. doi: 10.3390/biomedicines9121832 (PMC8698429; doi:10.3390/biomedicines9121832)
Supplement: Supplementary file 1 [file biomedicines-09-01832-s001.zip › biomedicines-1476591-supplementary.pdf]

Table S1. Primers and probes for genotyping of interbred mice with combined deficiencies of LDLR, Apobec, and Pg

Gene	Primer identification[a]	Sequence of oligonucleotide
Ldlr	WT-Ldlr-F	5’- CAAGACGTGCTCCCAGGATGACTTC
	WT/N-Ldlr-R (common)[a]	5’-CTTGTCCTTGCAGTCTGCCTCGCC
	WT-Ldlr-FITC[b]	5’-CAATCTCGGTCTCCATCACACAC-FITC
	WT-Ldlr-R640[c]	5’-R640-ACTGCGGGGAGATGCACTTGCCATC-P
	Null-Ldlr-NEO	5’-GATTGGGAAGACAATAGCAGGCATGC
	Null-Ldlr FITC[b]	5’-GCTGGTTCTTTCCGCCTCAGAA-FITC
	Null-Ldlr-R705[c]	5’-R705-CATAGAGCCCACCGCATCCCCA-P
Apobec1	WT-Apobec1-F	5’-GCCACTATGCCCAGGTCA
	WT/N-Apobec1-R (common)[a]	5’-CTCCAATACATACAGTTTCACCCAC
	WT-Apobec1-FITC[b]	5’-TTAGAGTATTGTTACTGCTGGAGGAA-FITC
	WT-Apobec1-R640[c]	5’-R640-TCGTCAACTACCCCCCTCTTCAAACG-P
	Null-Apobec1-NEO	5’-ACAAGCAAAACCAAATTAAGGGCCA
	Null-Apobec1-FITC[b]	5’-TGCTGATCTCGTTCTTCAGGCTAT-FITC
	Null-Apobec1-R705[c]	5’-R705-AACTGACACATTTGGAAACCACAG-TACTTAGAACCAC-P
Pg	WT-Pg-F	5'-TCAGCAGGGCAATGTCACGG
	WT-Pg-R	5'-CTCTCTGTCTGCCTTCCATGG
	WT-Pg-FITC[b]	5'-TCACACGGTCACCCAACACA-FITC
	WT-Pg-R640[c]	5’-R640-AGCATCAAAACAGGTGCGGCA-P
	Null-Pg-F	5’-GACATGGATGGCTGAACCGTCTCTGT
	Null-Pg-R	5'-ACAAGCAAAACCAAATTAAGGGCCA
	Null-Pg-TAMURA/BHQ[d]	5’-TAMRA-CATAGCCTGAAGAACGAGATCA-GCAGCC-BHQ2

[a] Primer used for both WT and null (N) alleles. F, forward; R, reverse.

[b] Donor FRET probe.

[c] Phosphorylated (P) acceptor FRET probes.

[d] TAMURA/BHQ probe.
